# Baseline Pupil Diameter Is Not a Reliable Biomarker of Subjective Sleepiness

**DOI:** 10.3389/fneur.2019.00108

**Published:** 2019-02-25

**Authors:** Inès Daguet, Didier Bouhassira, Claude Gronfier

**Affiliations:** ^1^Lyon Neuroscience Research Center, Waking Team, INSERM UMRS 1028, CNRS UMR 5292, Université Claude Bernard Lyon 1, Université de Lyon, Lyon, France; ^2^INSERM U987, Centre d'Evaluation et de Traitement de la Douleur, Hôpital Ambroise Paré, Boulogne-Billancourt, France

**Keywords:** pupil diameter, sleepiness, circadian rhythm, sleep pressure, constant routine

## Abstract

Sleepiness is commonly seen as reflecting the basic physiological need to sleep and is associated with physiological and neurobiological changes. Subjective evaluations of sleepiness, however, are neither representative of cognitive and physical performances, nor of physiological sleepiness. Finding a simple, rapid, and objective marker of sleepiness is essential in order to prevent errors and accidents, but this has remained largely unsuccessful. The aim of this study was to determine whether the baseline pupil diameter is a physiological biomarker of sleepiness at all times of day and to isolate the regulatory components involved. Twelve healthy men (20–29 years old) participated in a 56-h experimental protocol, including a 34-h constant routine paradigm with enforced wakefulness. This protocol was used in order to eliminate the potential influence of all environmental rhythms and reveal the endogenous circadian rhythmicity of two physiological measures: sleepiness and pupil diameter. Sleepiness was assessed subjectively every hour on a computerized 10 cm visual analog scale and pupil size was recorded every 2 h with a hand-held video-pupilometer. Our results revealed that subjective sleepiness increased linearly with time awake and displayed a circadian rhythm. Baseline pupil diameter showed a linear decrease with time spent awake as well as a circadian 24-h rhythm. This is the first evidence of a circadian variation of the baseline pupil size in a highly-controlled constant routine paradigm conducted in very dim light conditions. An overall negative correlation between the size of the pupil and the subjective level of sleepiness was observed. Analyzing the contribution of the two sleep regulation components in this correlation, we further showed: (1) a negative correlation between the homeostatic sleep pressure components, (2) a negative correlation between the circadian drives only during half of the 24 hours, corresponding to 62% of the biological day and 25% of the biological night. These results highlight that, due to the dual regulation of sleepiness by the homeostatic and circadian processes, baseline pupil diameter is an index of sleepiness only at certain times and therefore cannot be used as a systematic and reliable biomarker of sleepiness.

## Introduction

Sleepiness reflects the basic physiological need to sleep, and is classically recognized by yawning, eye rubbing and nodding (1). These behavioral changes are generally associated with neurobiological correlates such as cognitive decrements, microsleep episodes and an increase in alpha and theta activity in the EEG signal (2). Sleepiness results from the combination of a homeostatic process and a circadian process (3, 4). The homeostatic drive (process S) increases with wakefulness and decreases during sleep. The circadian drive (process C) relies on the self-sustained rhythmic 24-h activity of the endogenous biological clock, located in the suprachiasmatic nucleus (SCN) of the hypothalamus. These two processes interact to control the timing of sleep and wakefulness. Interestingly, their seemingly paradoxical drive, i.e. the high homeostatic sleep pressure at the end of the day together with the concomitantly high circadian pressure promoting wakefulness, is crucial to consolidate wakefulness during daytime and sleep at night (4).

Monitoring sleepiness is crucial in order to prevent accidents in everyday life conditions; for example in monotonous jobs, when driving a vehicle, or during night work. Yet, the assessment of sleepiness is also essential in patients suffering from sleep disorders, in order to diagnose and monitor excessive sleepiness or to evaluate the efficiency of a treatment. Different technics based on the measurement of objective physiological responses such as heart rate, skin conductance, reaction time, sleep latency, or pupil variations have been tested to estimate the level of sleepiness (5, 6). In clinical practice, sleep tendency during the day is commonly assessed by two validated tests: the Multiple Sleep Latency Test (MSLT) and the Maintenance of Wakefulness Test (MWT). However, both tests take almost a day to be conducted and sometimes need to be scheduled months in advance in a sleep clinic. Therefore, the need for a faster and more convenient biomarker of sleepiness is undeniable (6).

The eye has been the target of numerous studies on sleepiness as the opening or closing of the eyelids is one of the major differences between the states of sleep and wake. Indeed, the first studies using pupillometry as a biomarker of sleepiness emerged in the 1950s (7–9) and several variables have been studied over the years (6, 10–12). For example, psychological pupillography has been used in order to measure the percentage of eyelid closure during a vigilance task (11). The Pupillary Unrest Index (PUI) of the Pupillographic Sleepiness Test (PST) has been used to detect spontaneous pupillary oscillations, by measuring infrared video pupillography in darkness (12). Similarly, baseline pupil diameters have been measured in darkness during wake and sleep (12, 13). At first sight, it seems that the baseline pupil diameter could be a good marker of sleepiness as the pupil is dilated during wake and constricted during REM sleep (13). During NREM sleep, pupil size oscillates between small and large pupil diameters reflecting sleep depth; the deeper the sleep, the more the pupil constricts.

The circuitry involved in the control of sleep engages the reticular activating system, which consists of several nuclei such as the ventral tegmental area, the raphe nuclei and the locus coeruleus (LC) (14, 15). Even though sleepiness is thought to be controlled by the same structures, the neural substrates involved have not been clearly identified. The parasympathetic drive has been shown to be higher during sleep stages than during relaxed wakefulness, suggesting an implication of the autonomic nervous system in sleepiness (16). Other mechanisms, such as adenosine have been proposed to participate in sleepiness and sleep pressure (17), by activating “sleep-active” neurons in the ventro-lateral preoptic area of the hypothalamus (VLPO).

Pupil diameter in constant darkness is regulated by the automatic nervous system; activation of the parasympathetic pathway induces pupil constriction whereas activation of the sympathetic pathway leads to pupil dilation (18). In everyday life, pupil size is strongly influenced by the ambient lighting. Light stimulates the classic photoreceptors (rods and cones) but also activates the recently discovered intrinsically photosensitive retinal ganglion cells (ipRGCs), also known as melanopsin-containing retinal ganglion cells. These ipRGCs axons constitute the retino-hypothalamic tract (RHT) and project, among other structures, to the olivary pretectal nucleus (OPN), a brain region involved in the control of the pupillary light reflex (PLR) (19).

In sleepy subjects who are in the dark, the pupil size decreases and large spontaneous oscillations, called sleepiness waves, appear (10, 20–23). The intensity of these sleepiness waves increases with the duration of sleep deprivation (24). On the opposite, in non-sleepy subjects, the pupil remains constantly dilated in darkness. When comparing the pupil diameter in sleep deprived and alert conditions in the same participants, the pupil size is significantly smaller in sleep deprivation conditions (25, 26). Similarly, a large pupil size is associated with high levels of cognitive effort (27). A relationship between pupil diameter and sleepiness has also been observed in pathologies such as narcolepsy, with narcoleptic patients, who suffer from excessive daytime sleepiness, showing smaller pupil diameters than healthy participants, both in ambient light and in darkness (28).

Despite the numerous studies that have examined the relationship between alertness and pupil diameter, discrepancies remain. Certain studies have identified an association between high sleepiness and small pupil size (20, 25, 27–29) whereas others have not observed such a correlation between the two responses, both for intra-individual (30) and inter-individual correlations (31). It is important to highlight that these pupillometry and sleepiness measures were conducted either in the morning, the afternoon, the evening, or at night, depending on the study. Surprisingly, and even though this could explain the disparity in the results, the time-of-day effect has rarely been taken into consideration, suggesting that the role of the circadian system, both in regulating sleepiness and possibly pupil diameter, has been forgotten in these analyses.

To the best of our knowledge, only three studies have looked at the time course of the pupil diameter over the 24 hours (23, 26, 32). Loving and collaborators (32) measured pupil diameter in healthy adults every 30 min during a 24-h episode of constant wakefulness (sleep deprivation protocol) in ambient red light (<100 lux) and showed no variation of pupil size, suggesting no relationship between sleep debt and pupil size. In a 27-h paradigm with an ultradian sleep/wake cycle (15 min nap opportunity every hour) with ambient light (80 lux), Ranzijn et al. (26) observed that the baseline pupil diameter became smaller with progressive sleep loss, but this variation was not correlated to subjective sleepiness. Wilhelm et al. (23) conducted a 30-h enforced wakefulness protocol in constant low light levels (2 cd/m^2^) with measures every 2 h, and revealed a decrease in pupil diameter and an increase in subjective sleepiness as time spent awake increased. Nevertheless, no studies have analyzed how sleepiness and pupil size covary as a function of time of day.

The objective of our study was to determine whether the baseline pupil diameter is a physiological biomarker of sleepiness at all times of day and to isolate the regulatory components involved (homeostatic and circadian). We used the highly controlled constant routine procedure in healthy individuals to separate and investigate how these two processes correlate with pupil diameter at different times of day. We hypothesized that the relationship between pupil diameter and sleepiness is not linear, simply related to the homeostatic drive for sleep, but more complex than previously thought, and involving the circadian system.

## Materials and Methods

### Participants

Twelve healthy men (20–29 years old, mean = 22.7 ± 3.3 years old; BMI = 21.8 ± 3.1 kg/m^2^) were included in this study. Neurological, psychiatric and sleep disorders were excluded by physical examination and psychological questionnaires (Pittsburg Sleep Quality Index Questionnaire and Beck Depression Inventory) (33, 34). Participants had an intermediate chronotype (Horne and Ostberg Chronotype Questionnaire score between 31 and 69) and did no shift work, nor transmeridian travel during the previous 3 months (35). Participants had normal visual acuity (Landolt Ring Test and Monoyer scale), contrast vision (Functional Acuity Contrast Test) and color vision (Farnworth D-15 and Ishihara Color Test). All experimental procedures were carried out in accordance to the principles of the Declaration of Helsinki. The study was approved by the local Research Ethics Committee (CPP Lyon Sud-Est II) and participants provided written informed consent.

### Study Design

**Figure 1:**
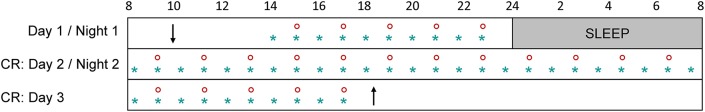
Overview of the experimental protocol. After a habituation day (day 1) and an 8-h sleep episode, participants underwent a 34-h constant routine (CR: days 2 and 3). Subjective sleepiness was measured every hour (blue star) and pupil size was recorded every 2 h (red circle). Participants arrived around 10:00 on day 1 (down arrow) and left the protocol around 18:00 on day 3 (up arrow).

Participants were instructed to maintain a regular sleep-wake schedule (bedtimes and waketimes within ± 30 min of self-targeted times) for 1–3 weeks before admission to the laboratory, and this was verified by wrist activity and light exposure recordings (ActTrust, Condor Instruments, São Paulo, Brazil). Subjects were then admitted to the laboratory for a 56-h experimental protocol (Figure 1) where they were maintained in an environment free from external time cues (clocks, television, smartphones, internet, visitors, sunlight, etc.). Subjects maintained contact with staff members specifically trained to avoid communicating time of day or the nature of the experimental conditions to the subjects. Participants arrived around 10:00 on the first day, they familiarized themselves with the laboratory environment, the low light levels (~0.5 lux), the equipment, and the measurements. A series of measurements were carried out until bedtime (participant's habitual bedtime), and an 8-h sleep episode was scheduled (constant darkness; recumbent position). This was followed by a 34-h constant routine protocol that started at the participant's usual waketime on day 2 and finished around 18:00 on day 3.

### Constant Routine Protocol

A Constant Routine (CR) paradigm was used in order to reveal the endogenous circadian rhythmicity of different parameters. The CR is conducted under constant environmental conditions, in order to eliminate or distribute across the circadian cycle the physiological responses evoked by environmental or behavioral stimuli (i.e., sleeping, eating, changes in posture, light intensity variations) (36, 37). In practical terms, participants were asked to remain awake for 34 h with minimal physical activity, while lying in a semi-recumbent (45°) posture in bed. This posture was also maintained for urine samples and bowel movements. Room temperature (mean = 23°C ± 0.6) and ambient very dim halogen light remained constant. Light intensity was homogeneous in the room (~0.5 lux at the participant's eye level in all gaze directions). Participants were given small equicaloric snacks and fluids at hourly intervals, to maintain an equal nutritional caloric intake and stable hydration across the circadian cycle. Caloric requirements were calculated with use of the Wilmore nomogram to determine the basal metabolic rate and were adjusted upward by a 7% activity factor (38, 39). Fluid intake was calculated for each subject to account for the sedentary nature of the CR (38). A study staff member remained in the room with the participant at all times during the CR to monitor wakefulness and to ensure compliance to study procedures.

### Sleepiness Evaluation

Sleepiness was assessed subjectively every hour on a computerized 10 cm visual analog scale (VAS) ranging from 0 (no sleepiness) to 10 (maximum sleepiness).

### Pupillometry

Pupil size was recorded every 2 h with a hand-held monocular video-pupilometer (Neurolight, IDMed, Marseille, France). This device, placed at 25 mm from cornea surface, detected pupil margins under infrared illumination (two infrared LED lights with a peak at 880 nm) and continuously tracked the pupil diameter. The pupilometer was placed in front of the participant's left eye and held steadily by the experimenter (Supplementary Figure 1). The participant was asked to keep the left eye wide open (without blinking) and to look straight ahead. During the measurement, the experimenter could see the pupil on the screen of the device and check that the device was correctly placed on the participant's eye. This measurement was conducted in complete darkness as the right eyelid was closed and covered by the participant's hand and the left eye was covered by the device. Before each measurement, we also questioned the participant in order to ensure that the participant did not detect any ambient lighting. The baseline pupil diameter was detected over a 5 s segment in darkness (without adaptation), with a sampling rate of 62 Hz and was determined by calculating the median pupil diameter during the stable portion of this 5 s measurement. Pupil diameter was recorded in mm in the output file of the pupilometer. Pupil diameter was considered abnormal when values were above 9 mm or below 2 mm. Artifacts were defined when an absolute change between 2 samples (sampling rate of 62 Hz) was above 0.15 mm (which corresponds to a change of approximatively 9.3 mm per second).

### Data Analysis

The sleepiness and pupil size measurements conducted during the first 2 h after waketime were removed from all analyses because sleep inertia has been shown to impair physiological responses such as alertness and cognitive function (40, 41). An outlier test was also applied on raw sleepiness and pupil diameter data, which identified no outliers in the datasets. To reduce inter-individual variability, all data were normalized by calculating individual z-scores (except for the analysis on raw values described in the Supplementary Material). For temporal analysis all values were plotted (32 values for sleepiness and 16 values for pupil size). For correlations, only half of the sleepiness values were used (measure every 2 h, starting 3 h after waketime), in order to have the same number of points for sleepiness and pupil size. After verification that data were normally distributed (Shapiro-Wilk test), Pearson correlations were computed between pupil diameter and sleepiness scores collected over the 34-h constant routine. To model the effect of time on the responses, the two main components regulating sleep (process S and process C) were modeled on the 34-h constant routine dataset. The homeostatic component (process S) of the data was regressed by a linear model on z-score transformed values (after removal of the sinusoidal trend): *f*(*time*) = *y*0 + *a* × *time*. Circadian rhythmicity (process C) was determined on linearly detrended z-score values using a sinusoidal regression model: $f\left(time\right)=mesor+amplitude\times \text{cos}\left(2\pi \times \frac{\text{time}}{\text{tau}}+\text{phase}\right)$; Tau (circadian period) was constrained between 23.5 and 24.5 h (42, 43). Statistics were computed with *R* (Version 3.5.1, 2018-04-23, R Foundation for Statistical Computing, Vienna, Austria) and *SigmaPlot* (Version 12.0, Systat Software, San Jose, CA). Results were considered significant for *p* < 0.05.

## Results

### Correlation Between Baseline Pupil Diameter and Subjective Sleepiness

**Figure 2:**
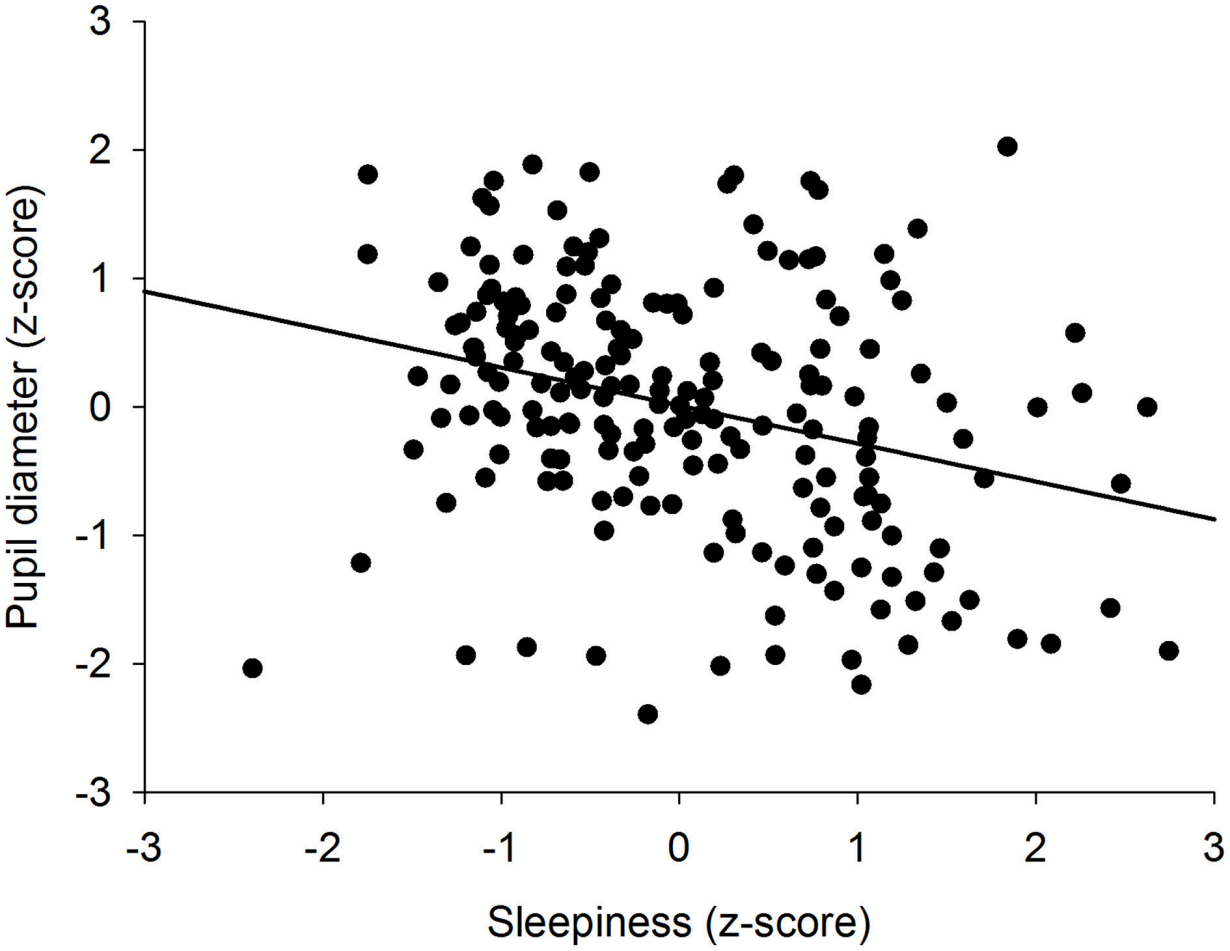
Correlation between individual baseline pupil diameter and individual subjective sleepiness (*R*^2^ = 0.09; *p* ≤ 0.0001).

**Figure 3:**
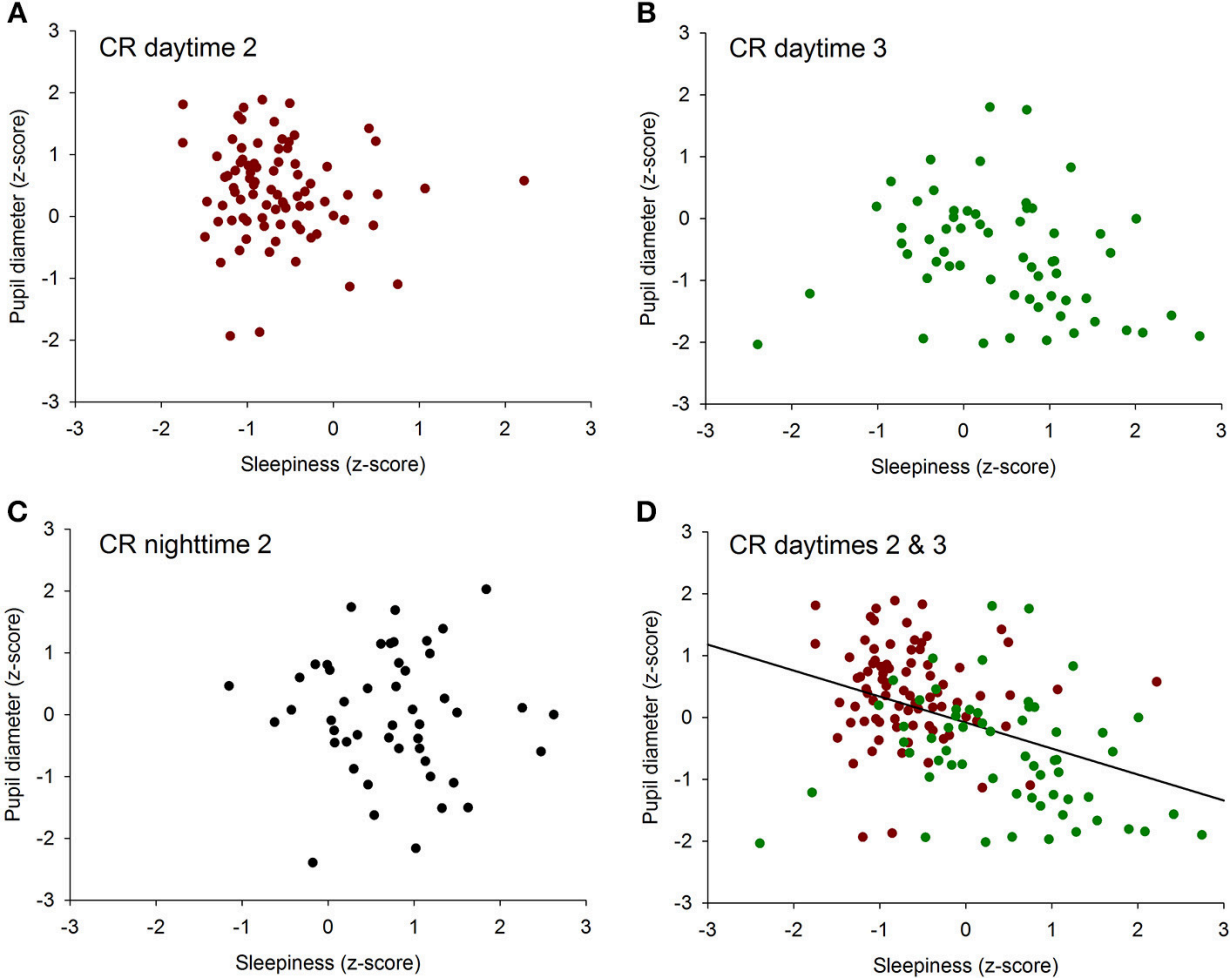
Correlation between individual baseline pupil diameter and individual subjective sleepiness. **(A)** CR daytime 2 (first 16 h of the constant routine protocol), no correlation (*p* = 0.34). **(B)** CR daytime 3 (last 10 h of the protocol), no correlation (*p* = 0.07). **(C)** habitual nighttime, no correlation (*p* = 0.78). **(D)** habitual daytime (CR daytime 2 and CR daytime 3 pooled together), negative correlation (*R*^2^ = 0.17; *p* ≤ 0.0001).

To determine if there was an overall association between the size of the pupil and the level of subjective sleepiness during the 34-h constant routine protocol, all 16 values of all 12 participants were plotted on the same graph. The analysis on raw values revealed no correlation between subjective sleepiness and pupil diameter (*p* = 0.23; Supplementary Figure 2). In order to reduce inter-individual variability, all future analyses were conducted on normalized data (z-score calculation). Indeed, the negative correlation between pupil diameter and sleepiness is illustrated on Figure 2; the higher the sleepiness level, the lower the pupil diameter (*R*^2^ = 0.09; *p* ≤ 0.0001; Figure 2). In order to separate the potentially different relationships between pupil size and sleepiness during daytime and during nighttime, data were segregated into 4 time-episodes: CR daytime 2 (first 16 h of the constant routine protocol, corresponding to habitual daytime), CR nighttime 2 (next 8 h of the protocol, corresponding to habitual nighttime), CR daytime 3 (last 10 h of the protocol, corresponding to the first 10 h of habitual daytime after a full night of sleep deprivation) and CR daytimes 2 and 3 combined (habitual daytime over 2 days). The same correlation analysis was conducted on each of these epochs. No correlations between pupil size and sleepiness were observed for CR daytime 2, CR nighttime 2, nor CR daytime 3 respectively (Figures 3A–C respectively). When CR daytime 2 and CR daytime 3 were pooled together, a negative correlation appeared, suggesting that the time of day is a factor that needs to be taken into consideration (*R*^2^ = 0.17; *p* ≤ 0.0001; Figure 3D).

### Effect of Time-of-day on Subjective Sleepiness

**Figure 4:**
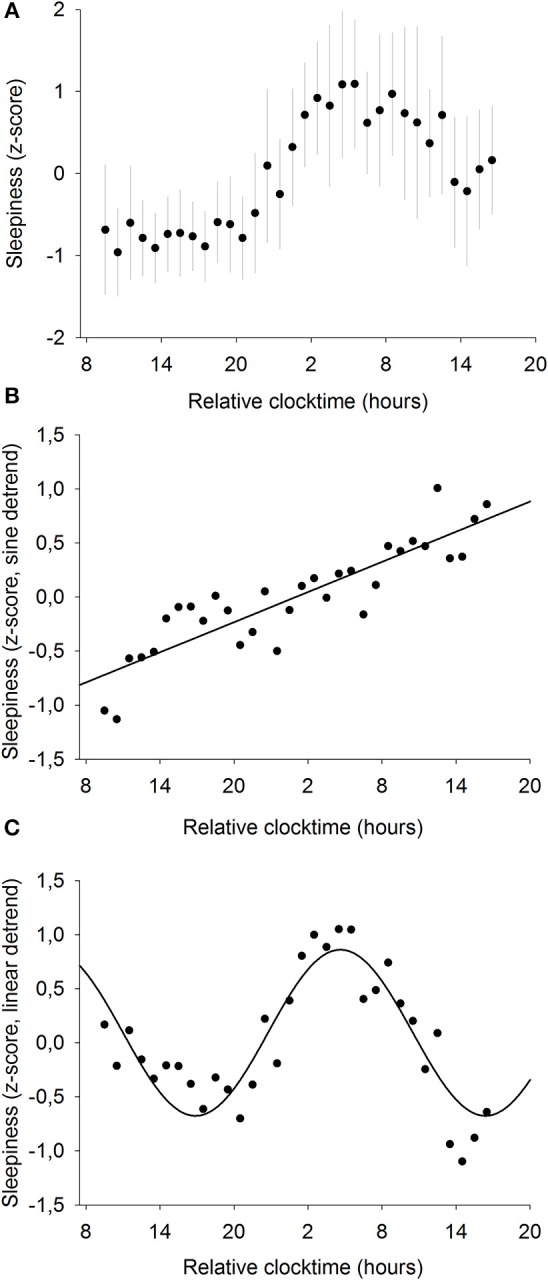
Mean subjective sleepiness (*n* = 12). **(A)** normalized data (mean ± SD). **(B)** linear regression, after removal of the sinusoidal trend. Sleepiness increases with time spent awake (*R*^2^ = 0.78; *p* ≤ 0.0001). **(C)** sinusoidal regression, after removal of the linear trend; (*R*^2^ = 0.80; *p* ≤ 0.0001).

To further investigate the relationships between sleepiness and baseline pupil diameter, we analyzed the mechanisms responsible for their respective time course. Sleepiness was evaluated subjectively every hour during the 34-h constant routine (Figure 4A) and raw sleepiness values ranged from 0 to 10 cm with a mean of 3.6 ± 2.6 cm on the VAS. Two models were fitted on the data in order to model the two components of sleep regulation: a linear trend modeling homeostatic sleep pressure and a sinusoidal component modeling the circadian drive. First, a significant linear fit was observed, confirming that sleepiness increases with time spent awake (*R*^2^ = 0.78; *p* ≤ 0.0001; Figure 4B). Second, after removal of the homeostatic trend, a sinusoidal regression significantly modeled the data, with a peak of sleepiness at 04:30 and a trough at 16:30 (*R*^2^ = 0.80; *p* ≤ 0.0001; Figure 4C).

### Effect of Time-of-day on Baseline Pupil Size

**Figure 5:**
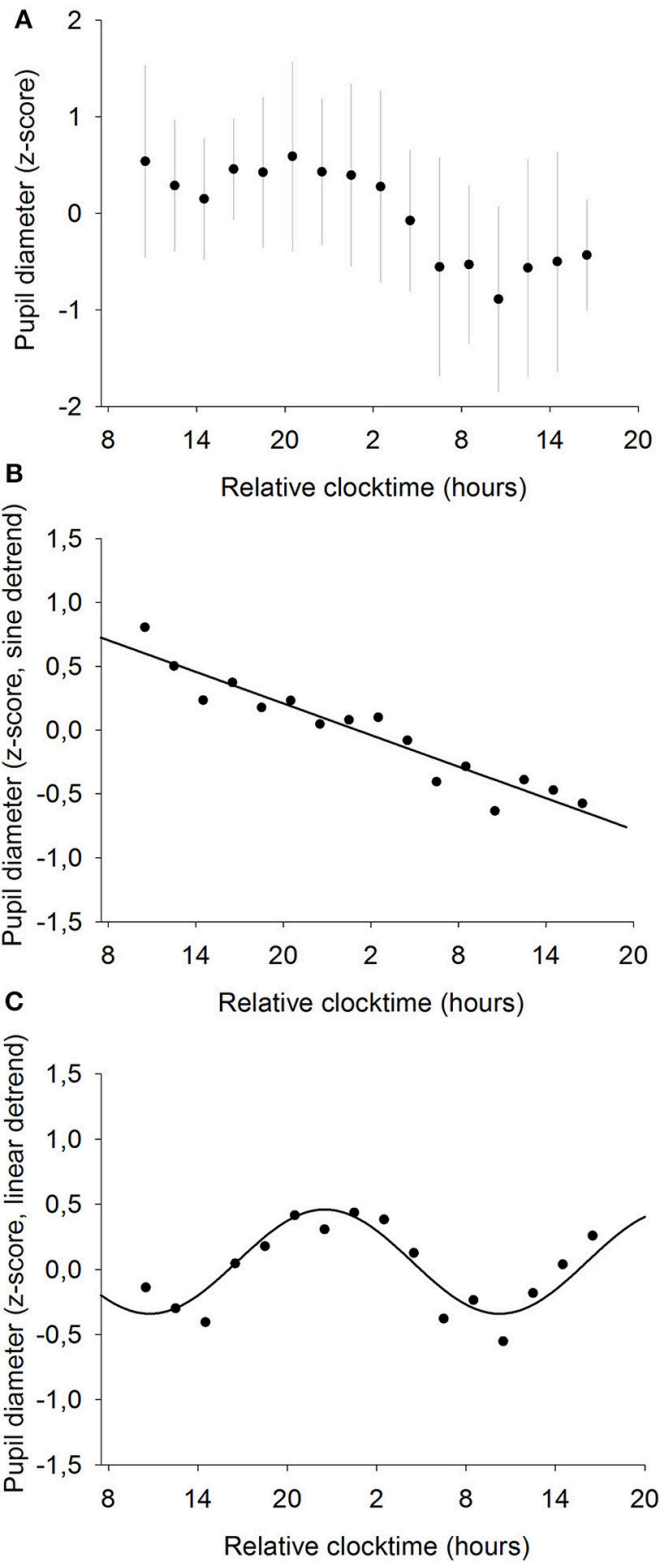
Mean baseline pupil diameter (*n* = 12). **(A)** normalized data (mean ± SD). **(B)** linear regression, after removal of the sinusoidal trend. Pupil size decreases with time spent awake (*R*^2^ = 0.69; *p* ≤ 0.0001). **(C)** sinusoidal regression, after removal of the linear trend (*R*^2^ = 0.91; *p* ≤ 0.001).

Pupil diameter was measured every 2 h throughout the whole 34-h constant routine (Figure 5A) and raw pupil size values ranged from 5.8 to 8.7 mm with a mean of 7.4 ± 0.7 mm. A statistically significant linear regression was found between pupil diameter and time elapsed since waketime (*R*^2^ = 0.69; *p* ≤ 0.0001; Figure 5B). Pupil size decreases as time spent awake increases. Moreover, a statistically significant sinusoidal fit was found on linearly detrended data, with the largest pupil size at 22:30 and the smallest at 10:30 (*R*^2^ = 0.91; *p* ≤ 0.001; Figure 5C).

### Separation of the Two Sleep Regulatory Components: Process S and Process C

**Figure 6:**
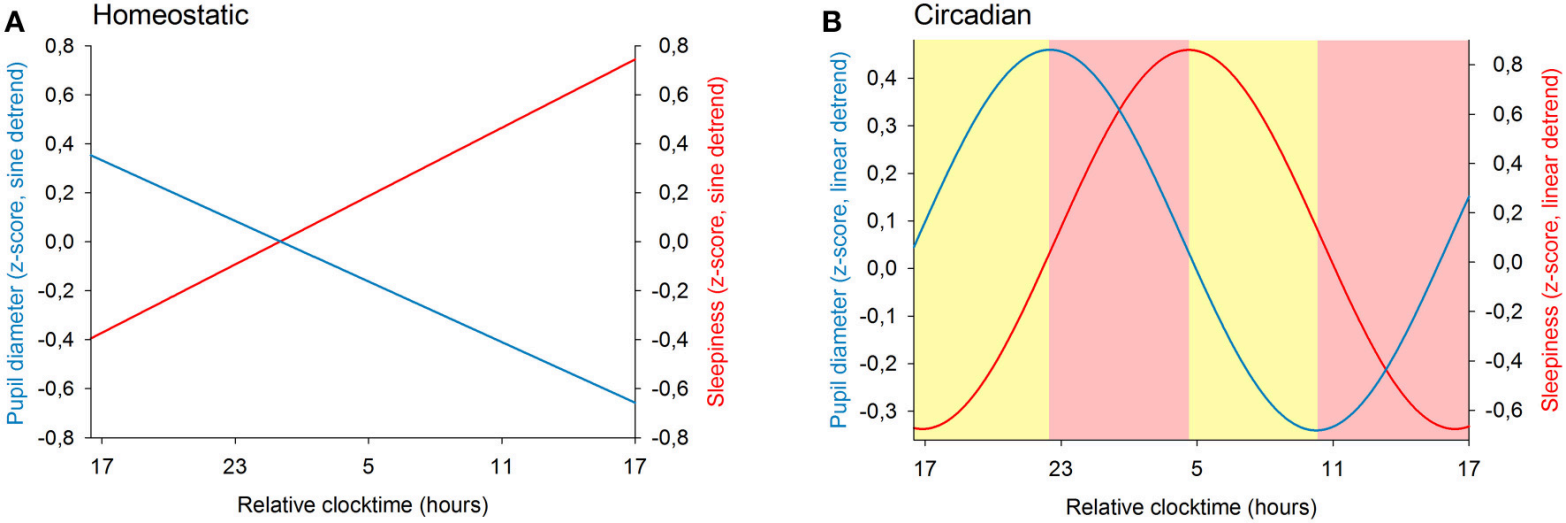
Modeling of the two components of sleep regulation on baseline pupil diameter (blue line) and on sleepiness (red line) illustrated over a 24-h segment. **(A)** linear models of the effect of homeostatic sleep pressure on pupil size and sleepiness (after sinusoidal detrend). **(B)** sinusoidal components modeling the circadian drive on pupil size and sleepiness (after linear detrend). The yellow shaded areas correspond to the episodes of time where the circadian components of pupil diameter and of sleepiness vary in the same direction. The pink shaded areas correspond to the episodes of time where the responses change in opposite directions.

**Figure 7:**
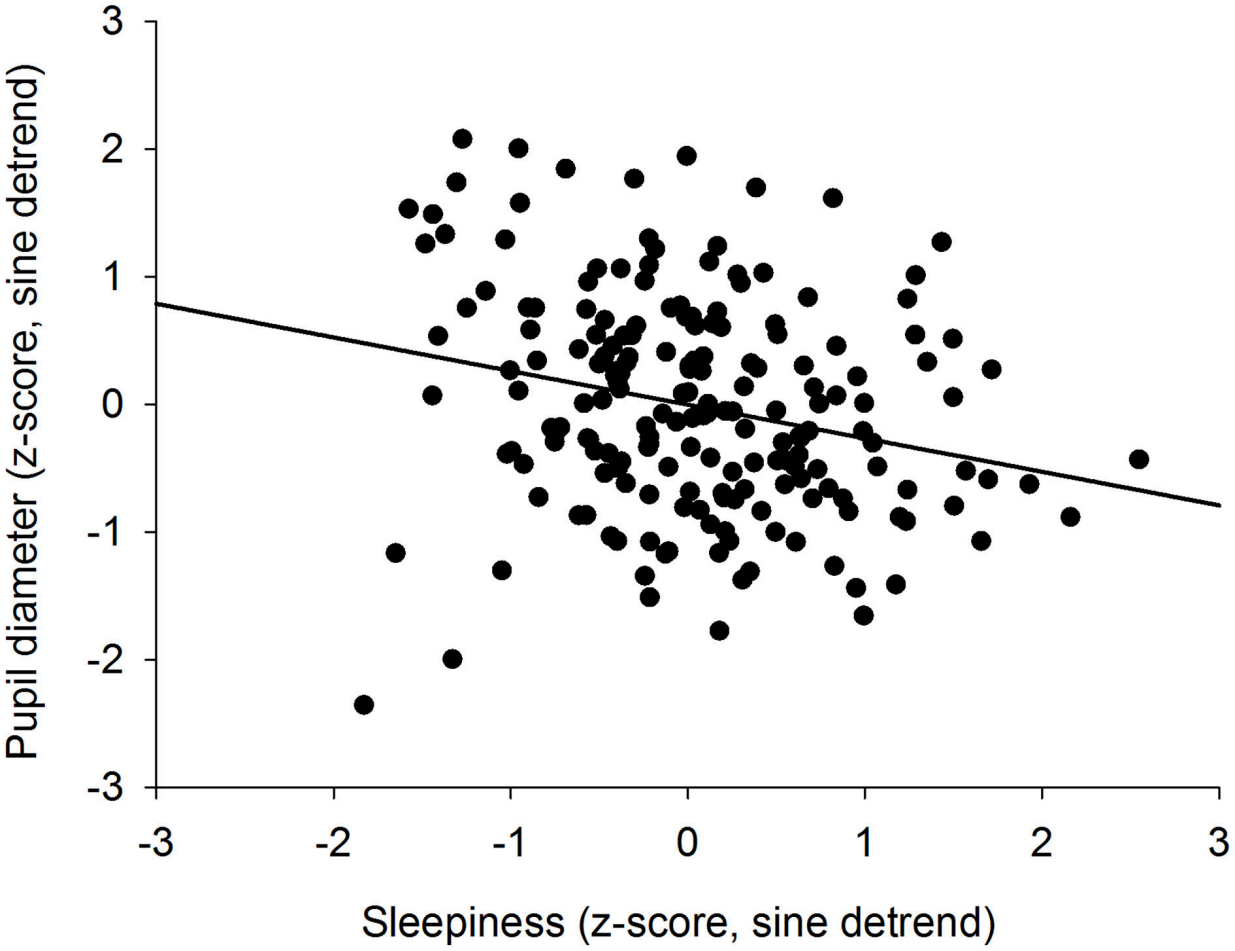
Correlation between the homeostatic components of pupil diameter and subjective sleepiness (*R*^2^ = 0.06; *p* ≤ 0.001).

**Figure 8:**
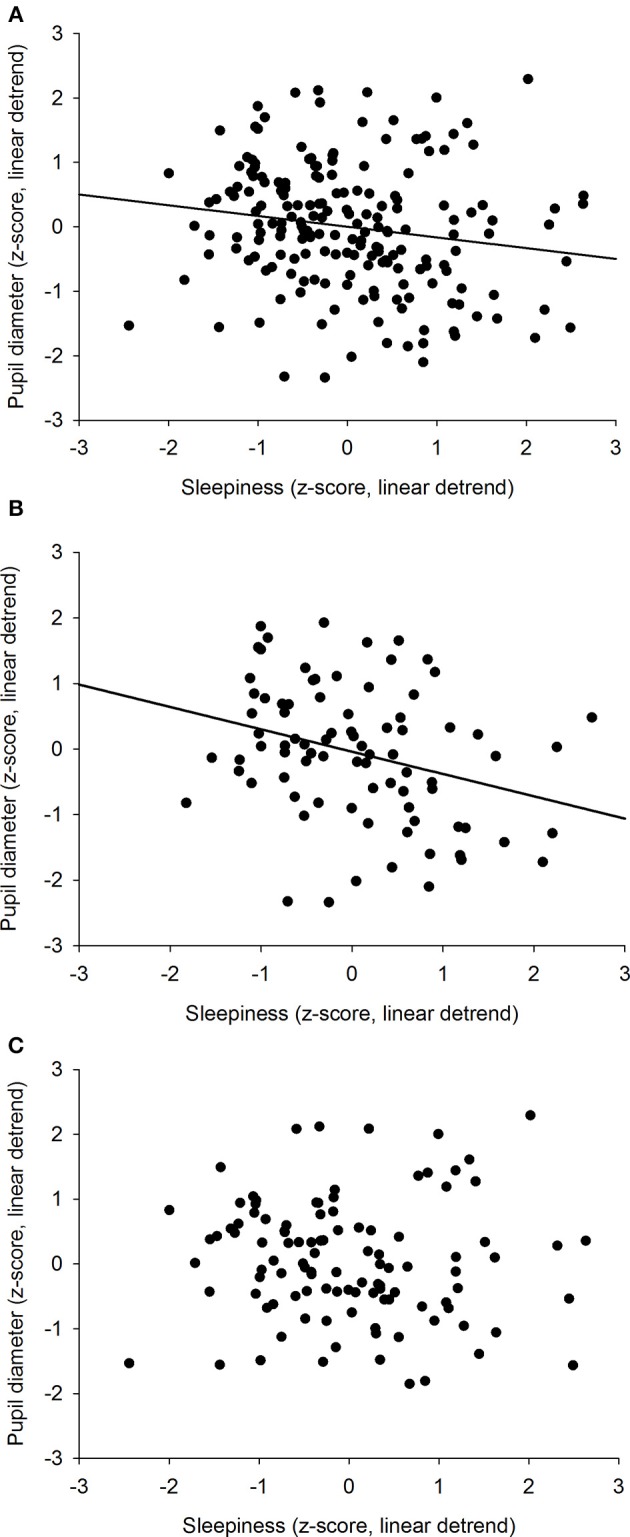
Correlation between the circadian components of pupil diameter and subjective sleepiness. **(A)** all values across the 34-h constant routine segment (*R*^2^ = 0.03; *p* ≤ 0.02). **(B)** values when 2 curves vary in the same direction (between 4:30 and 10:30 and between 16:30 and 22:30; *R*^2^ = 0.09; *p* ≤ 0.005). **(C)** values when 2 curves vary in opposite directions (between 10:30 and 16:30 and between 22:30 and 4:30; *p* = 0.57).

We previously showed that the time course of sleepiness and pupil size are influenced by two components (homeostatic sleep pressure and circadian variation). We have isolated each component in order to observe, on one hand, the correlation between the linear processes of sleepiness and pupil size (Figure 6A) and, on the other hand, the correlation between the sinusoidal processes of these responses (Figure 6B). First, the homeostatic models of sleepiness and pupil size, illustrated on Figure 6A, showed that these responses covaried linearly but negatively. The pupil diameter decreased with time awake whereas sleepiness increased, partly explaining the negative correlation between the two responses observed in Figure 2. Indeed, we found a significant negative correlation between the linear processes of sleepiness and pupil size, revealing that sleepiness was high when the pupil size was small (*R*^2^ = 0.06; *p* ≤ 0.001; Figure 7). Second, Figure 6B showed that the circadian drives of sleepiness and pupil diameter did not covary in phase (maximum pupil size at 22:30 and maximum sleepiness at 04:30.), with a 6-h phase-lag between the two rhythms. This lag allowed us to identify two different segments of time over the 24 hours: (1) the times of day when both responses vary in the same direction (both decrease or both increase) and (2) the times of day when they vary in opposite directions (one decreases when the other increases). The correlation between the circadian drives were therefore analyzed over the entire constant routine and during these two segments of time. We found a negative correlation between pupil size and sleepiness over the whole 24 hours (*R*^2^ = 0.03; *p* ≤ 0.02; Figure 8A) and also specifically when the two curves varied in the same direction (yellow shaded areas; between 4:30 and 10:30 and between 16:30 and 22:30; *R*^2^ = 0.09; *p* ≤ 0.005; Figure 8B). However, no correlation was found when they varied in opposite directions (pink shaded areas; between 10:30 and 16:30 and between 22:30 and 4:30; Figure 8C). These results demonstrate the presence of an association between the circadian drives for pupil size and sleepiness only during half of the 24-h day (in the morning and in the evening), or during 62% of the biological day and 25% of the biological night.

## Discussion

Our results show that the correlation between the size of the pupil and the subjective level of sleepiness is not systematic and that the pupil diameter cannot be a simple proxy for sleepiness. Sleepiness increases linearly as time spent awake increases and displays a circadian rhythm across the constant routine protocol. Pupil diameter shows a linear decrease with time spent awake, superimposed to a circadian 24-h rhythm. The separation of the two sleep regulatory processes (process S and process C) revealed that both homeostatic (S) processes correlate negatively. However, focusing the analysis on circadian (C) processes showed that the negative correlation between the two responses only appears at the beginning and the end of the biological day, but not in the afternoon or during the biological night.

### Time-of-day Effect on Subjective Sleepiness

The subjective level of sleepiness increases as time spent awake and sleep pressure increases. This linear relationship between sleep pressure and subjective sleepiness (evaluated by the Stanford Sleepiness Scale or SSS) had already been identified in previous studies (23, 24). This homeostatic regulation of sleep (process S) could be explained by the accumulation of adenosine during wakefulness (17). A circadian variation of sleepiness (process C), modeled by a sinusoid regression, has also been identified, with a peak of sleepiness at 4:30. These data confirm previous results showing the existence of a 24-h rhythm of sleepiness (evaluated by the Karolinska Sleepiness Scale or KSS), and suggesting its control by the human endogenous circadian clock, located in the SCN (44, 45).

### Time-of-day Effect on Baseline Pupil Diameter

We found that the size of the pupil showed a linear trend with time awake, suggesting that it is linked to the homeostatic increase in sleep pressure that occurs during the day. Therefore, the higher the sleep pressure, the smaller the pupil diameter. This result is in line with the study of Wilhelm et al., who showed a linear decreasing trend in pupil diameter in constant semi-darkness during a 30-h forced wakefulness protocol (23). Likewise, Ranzijn and collaborators showed that the pupil diameter is smaller after a 27-h constant routine protocol in constant ambient lighting (80 lux), than before the protocol (26). In our study, the constant routine protocol, conducted in highly controlled laboratory conditions, also allowed the identification of a circadian rhythm of the pupil size. This is the first evidence that the baseline pupil diameter in constant very dim-light conditions follows a 24-h rhythm in mammals (peak at 22:30) and that the origin of this rhythm is endogenous, and likely controlled by the SCN. One of the pioneer studies, in 1951, showed that the pupil diameter was not constant across the day with measures every 3 h (46). In 1998, Wilhelm and collaborators performed two pupil size measurements in constant darkness (82 s baseline) and found no time-of-day effect: no significant difference between the size of the pupil in the morning vs. afternoon (21). This lack of difference between morning and afternoon pupil diameter could be explained by the combination of a small sample size (*n* = 7) and of an insufficient sampling rate (only 2 measures). Zele et al. did not observe a significant variation of baseline pupil diameter with circadian time (10 s baseline), despite the hourly measures and the control of a number of environmental cues. The endogenous oscillation might have been masked by the lighting environment in 10 lux (compared to ~0.5 lux in our study) (47). On the opposite, a time-of-day effect of pupil size was shown by Kraemer et al. by measuring pupil diameter for 10 min every 2 h (after 2 min of dark adaptation) during a 16-h protocol (48). A few years later, Eggert et al. measured pupil size at two time points (once in the morning and once in the afternoon; 82 s baseline) on a large number of participants and they observed a smaller pupil diameter in the afternoon compared to the morning in constant darkness (12). Overall, the literature is not entirely consensual on the existence of a time-of-day effect on pupil diameter. This could be explained by the methodological differences in the protocols, such as ambient lighting ranging from darkness in some studies to relatively strong light (100 lux) in others, and/or the duration of the baseline pupil diameter measurement (values ranging from a few seconds to several minutes).

### Correlation Between Subjective Sleepiness and Baseline Pupil Size

Sleepiness increased linearly whereas pupil diameter decreased linearly with time spent awake, revealing an overall negative correlation between the two responses. This correlation between pupil size and sleepiness was previously shown by a number of authors (20, 23, 25, 28, 29). Similarly, an association between pupil size and vigilance states (evaluated by measuring response times) has also been observed (27). However, other experiments with frequent pupillary measures did not find this association (24, 26, 30). The separation of the data according to the time of day (daytime = CR daytimes 2 & 3; nighttime = CR nighttime 2) revealed a correlation between the two responses during daytime only. This analysis allowed us to observe the changes in the relationship between pupil size and sleepiness, identifying for the first time that the pupil is a marker of sleepiness only at certain segments of the 24-h rhythm and therefore suggesting that their association is not solely linear.

### Pupil Size as a Marker of Homeostatic (Process S) and Circadian (Process C) Sleepiness

The results of our study show that sleepiness and pupil size have opposite linear trends, and reveal that they also follow a 24-h rhythm. The linear trends suggest that the homeostatic component of pupil size could be a marker of homeostatic sleepiness and the 24-h rhythms suggest that circadian variation of pupil size could be a marker of circadian sleepiness. However, the peak of the circadian variation of sleepiness happens around 04:30, whereas the maximum pupil size is observed at 22:30, suggesting that the two circadian rhythms are phase-lagged by approximately 6 h. This large phase difference excludes a causal relationship between circadian sleepiness and circadian pupil size and suggests that they do not drive each other but that they are controlled by separate pathways, both depending on the circadian system. This phase-lag phenomenon between circadian rhythms is not unknown, and is in fact even classical. As an example, although body temperature and cortisol secretion are both driven by the circadian system, their rhythms are not in phase; while cortisol release peaks around habitual waketime, body temperature peaks 9 hours later (49).

The specific time epoch correlation analyses we have conducted according to circadian rhythmicity (signals varying in the same or in opposite directions) invalidate the use of pupil diameter as a reliable biomarker of sleepiness. Indeed, the fact that sleepiness and pupil size are not driven at the same circadian phase, makes pupil size a correlate of sleepiness only at certain times of the day (morning and evening, corresponding to 50% of the 24 hours,) and not at others. Along this line, a 24-h variation in the pupillary light reflex has been shown and interpreted as evidence that ipRGC activity is driven by the circadian system (47, 50). Given our results, the 24-h rhythm of pupil constriction should not be considered as a “pure” marker of circadian variation of ipRGC sensitivity to light.

### Neurobiological Bases

Whereas the pupillary light reflex (PLR) depends on the activity of retinal photoreceptors (rods, cones and ipRGCs), the baseline pupil diameter in darkness is exclusively regulated by the autonomous nervous system (15, 22, 51). Wakefulness is associated with a large pupil size whereas during REM sleep small pupil sizes are observed (13). During anesthesia, a progressive dilation of the pupil indicates a greater loss of consciousness and a deepening of the anesthesia for certain anesthetics (18), however this has not been observed with other anesthetics, such as isoflurane (52). This result is in agreement with the literature and the results of our study, which show that overall as sleepiness increases, the pupil becomes smaller. Pupil dilation is known to originate from an activation of the sympathetic pathway or an inhibition of the parasympathetic circuitry (18). As it has been described by Samuels and collaborators (15, 53), an activation of the Locus Coeruleus (LC), conveyed to the VLPO, results in an increase in EEG signs of alertness, and a decrease in sleepiness. In parallel, an increased LC activity induces an increase in sympathetic activity and a decrease in parasympathetic activity, resulting in an increase in pupil diameter. This pupil dilation is mediated by the LC-Edinger-Westphal Nucleus (EW) pathway (15, 53). This dual circuitry shows that the LC influences both sleepiness and pupil diameter. This is in line with the results of Murphy and collaborators, who showed that there is a positive correlation between the pupil size and the BOLD signal in the LC (54). Therefore, we hypothesize that as sleepiness increases during the day, with the accumulation of homeostatic sleep pressure, the LC firing decreases, also inducing a decrease in pupil diameter. In this case the absence of stimulation of the fibers from the LC and the A1-A5 nuclei of the brainstem desinhibits the EW, which in turn activates the descending parasympathetic pathway, resulting in a pupil constriction (12, 15, 22). However, as we have seen previously, baseline pupil diameter and sleepiness do not simply vary linearly, as both of these responses also show a circadian rhythm, suggesting an interaction between the SCN and the LC. In this line, a circadian variation of the firing of the LC neurons has been observed in rats placed in constant darkness, with a faster firing rate during the active phase than during the inactive phase (55). Similarly, Takahashi et al. showed that in mice the noradrenergic neurons of the LC have a higher discharge rate during active wake compared to quiet wake, confirming that the LC is a wake-promoting structure (56). Here, we could hypothesize that the SCN regulates the LC activity, activating the LC neurons during the day and inactivating them during the night.

### Limitations

This study exposes a few limitations. Firstly, the population examined in this study is only composed of men. However, we do not expect different results in women as no gender effect on pupillometry measures was observed by Eggert et al. on a large population (12). Similarly, although the amplitude of the circadian drive might be slightly more important in women vs. men, sleepiness has never been shown to be driven by different mechanisms (57). Secondly, the duration of the baseline pupil measurement was only 5 s, and one might think that a longer duration would be preferable. We do not think that our short segment is a problem, as the baseline measurements were very stable across and within subjects (mean *SD* < 0.05 mm), and ranged from (5.8 to 8.7 mm) which does not differ from those of previous studies (23, 25). Thirdly, even though we believe this is highly unlikely, we cannot exclude that exposure to very dim light intensity of ~0.5 lux may have had an effect on the pupil size subsequently measured in darkness. Fourthly, this protocol was conducted in an extremely controlled environment which does not reflect real life conditions, such as light exposure. Indeed, it is well known that light increases vigilance, decreases sleepiness and decreases pupil size (10, 58), suggesting that in real life conditions, the effect of light on the pupil could mask the effect of sleepiness that is hoped to be observed (59, 60).

## Conclusion

Overall, our results show that even though baseline pupil diameter and sleepiness vary in opposite directions over the course of the day, their association is not that simple and is not solely the result of a homeostatic mechanism. The additional drive from the circadian timing system makes it more complex as it reveals an association only during half of the 24-h day, corresponding to 62% of the biological day and 25% of the biological night. These results demonstrate that due to the dual regulation of sleepiness by the homeostatic and circadian processes, baseline pupil diameter cannot be used as a reliable biomarker of sleepiness. Yet, finding an objective and convenient marker of sleepiness remains a priority as the subjective evaluation of sleepiness is not representative of performances or physiological sleepiness (20). Such a marker would be particularly useful to evaluate sleepiness in night workers, who are the highest at risk of making mistakes or injuring themselves (61, 62).

## Data Availability

The raw data supporting the conclusions of this manuscript will be made available by the authors, without undue reservation, to any qualified researcher.

## Author Contributions

The experiment was conceived by CG and designed by CG and ID. Data collection was performed by ID and CG. Data analyses were conducted by ID. ID and CG interpreted the data and wrote the manuscript. DB provided edits. All authors agreed to be accountable for all aspects of the work.

### Conflict of Interest Statement

The authors declare that the research was conducted in the absence of any commercial or financial relationships that could be construed as a potential conflict of interest.
