# Supplementary material for: Baseline Pupil Diameter Is Not a Reliable Biomarker of Subjective Sleepiness
Source: Front Neurol. 2019 Feb 25;10:108. doi: 10.3389/fneur.2019.00108 (PMC6398346; doi:10.3389/fneur.2019.00108)
Supplement: Supplementary file 1 [file Data_Sheet_1.pdf]

BASELINE PUPIL DIAMETER IS NOT A RELIABLE BIOMARKER OF SUBJECTIVE SLEEPINESS

Daguet I[1], Bouhassira D[2] and Gronfier C[1]
[1] Waking team, Lyon Neuroscience Research Center, Inserm UMRS 1028, CNRS UMR 5292, Université Claude Bernard Lyon 1, Université de Lyon, Lyon, France; [2] Inserm U987, Centre d'Evaluation et de traitement de la douleur, Hôpital Ambroise Paré, Boulogne-Billancourt, France

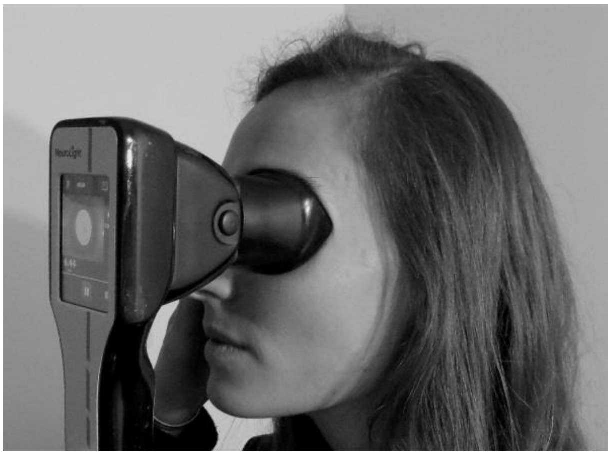

Supplementary figure 1: Hand-held monocular video-pupilometer device placed in front of the participant's eye for baseline pupil diameter measurement.

The data presented in the manuscript were normalized due to the high inter-individual variability. However, before this normalization procedure, the same analysis was conducted on raw data. This analysis revealed no correlation between raw pupil size and raw subjective sleepiness values (**Supplementary figure 2**). This absence of correlation has already been observed in experiments with frequent raw pupillary measures (1–3). After inter-individual variability was reduced by a normalization procedure a negative correlation was found; a large pupil size is associated with a low sleepiness level (**Figure 2 of main article**). The emergence of this correlation after normalization suggests that the correlation was hidden by the high inter-individual variability. This correlation between pupil size and sleepiness with normalized data (z-scores) was previously shown by Danker-Hopfe and collaborators (4). Similarly, an association between pupil size and vigilance states (evaluated with response times) has also been observed on normalized data (5). However, correlations have also been shown without normalizing the data (6–9).

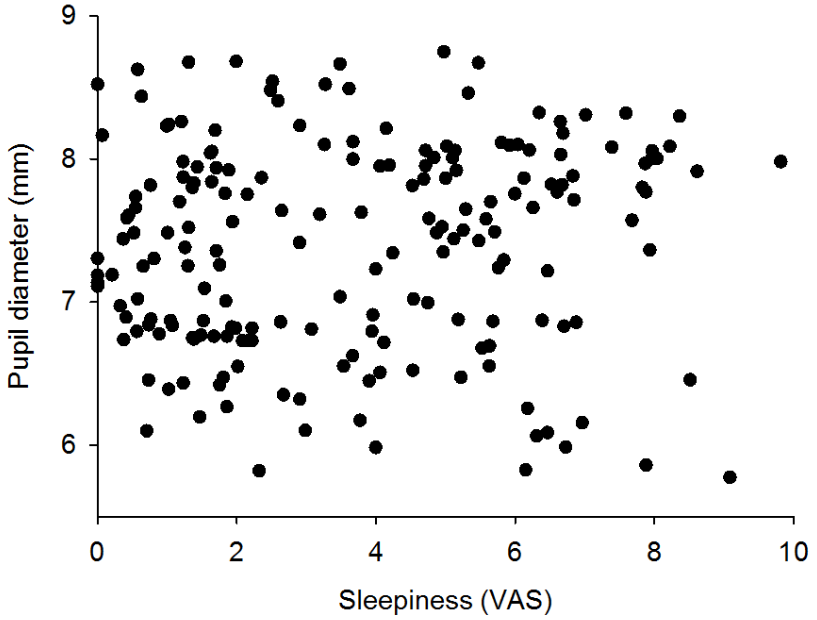

Supplementary figure 2: Absence of correlation between individual raw values of pupil diameter and subjective sleepiness (p=0.23).

References:

1. Wilhelm B, Wilhelm H, Lüdtke H, Streicher P, Adler M. Pupillographic assessment of sleepiness in sleep-deprived healthy subjects. *Sleep* (1998) **21**:258–265.
2. Ranzijn R, Lack L. The pupillary light reflex cannot be used to measure sleepiness. *Psychophysiology* (1997) **34**:17–22.
3. Lavie P. Ultradian rhythms in alertness - a pupillometric study. *Biol Psychol* (1979) **9**:49–62.
4. Danker-Hopfe H, Kraemer S, Dorn H, Schmidt A, Ehlert I, Herrmann WM. Time-of-day variations in different measures of sleepiness (MSLT, pupillography, and SSS) and their interrelations. *Psychophysiology* (2001) **38**:828–835.
5. Massar SAA, Lim J, Sasmita K, Chee MWL. Sleep deprivation increases the costs of attentional effort: Performance, preference and pupil size. *Neuropsychologia* (2018) doi:10.1016/j.neuropsychologia.2018.03.032
6. Morad Y, Lemberg H, Yofe N, Dagan Y. Pupillography as an objective indicator of fatigue. *Curr Eye Res* (2000) **21**:535–542. doi:10.1076/0271-3683(200007)2111-ZFT535
7. Yoss RE, Moyer NJ, Hollenhorst RW. Pupil size and spontaneous pupillary waves associated with alertness, drowsiness, and sleep. *Neurology* (1970) **20**:545–545.
8. Pressman MR, Spielman AJ, Korczyn AD, Rubenstein AE, Pollak CP, Weitzman ED. Patterns of daytime sleepiness in narcoleptics and normals: a pupillometric study. *Electroencephalogr Clin Neurophysiol* (1984) **57**:129–133.
9. Wilhelm B, Giedke H, Lüdtke H, Bittner E, Hofmann A, Wilhelm H. Daytime variations in central nervous system activation measured by a pupillographic sleepiness test. *J Sleep Res* (2001) **10**:1–7.
